# Supplementary material for: The concurrence of DNA methylation and demethylation is associated with transcription regulation
Source: Nat Commun. 2021 Sep 6;12:5285. doi: 10.1038/s41467-021-25521-7 (PMC8421433; doi:10.1038/s41467-021-25521-7)
Supplement: Supplementary file 1 — Supplementary Information [file 41467_2021_25521_MOESM1_ESM.pdf]

Supplementary information

The Concurrence of DNA Methylation and Demethylation is Associated with Transcription Regulation

Jiejun Shi[1], Jianfeng Xu[2], Yiling Elaine Chen[3], Jason Sheng Li[1], Ya Cui[1], Lanlan Shen[4], Jingyi Jessica Li[3], Wei Li[1*]

Affiliations:

[1]Division of Computational Biomedicine, Department of Biological Chemistry, School of Medicine, University of California, Irvine, Irvine, CA 92697, USA

[2]Department of Molecular and Cellular Biology, Baylor College of Medicine, Houston, TX 77030, USA

[3]Department of Statistics, University of California, Los Angeles, CA 90095, USA

[4]Department of Pediatrics, Baylor College of Medicine, USDA/ARS Children's Nutrition Research Center, Houston, Texas, 77030, USA

*Correspondence: wei.li@uci.edu

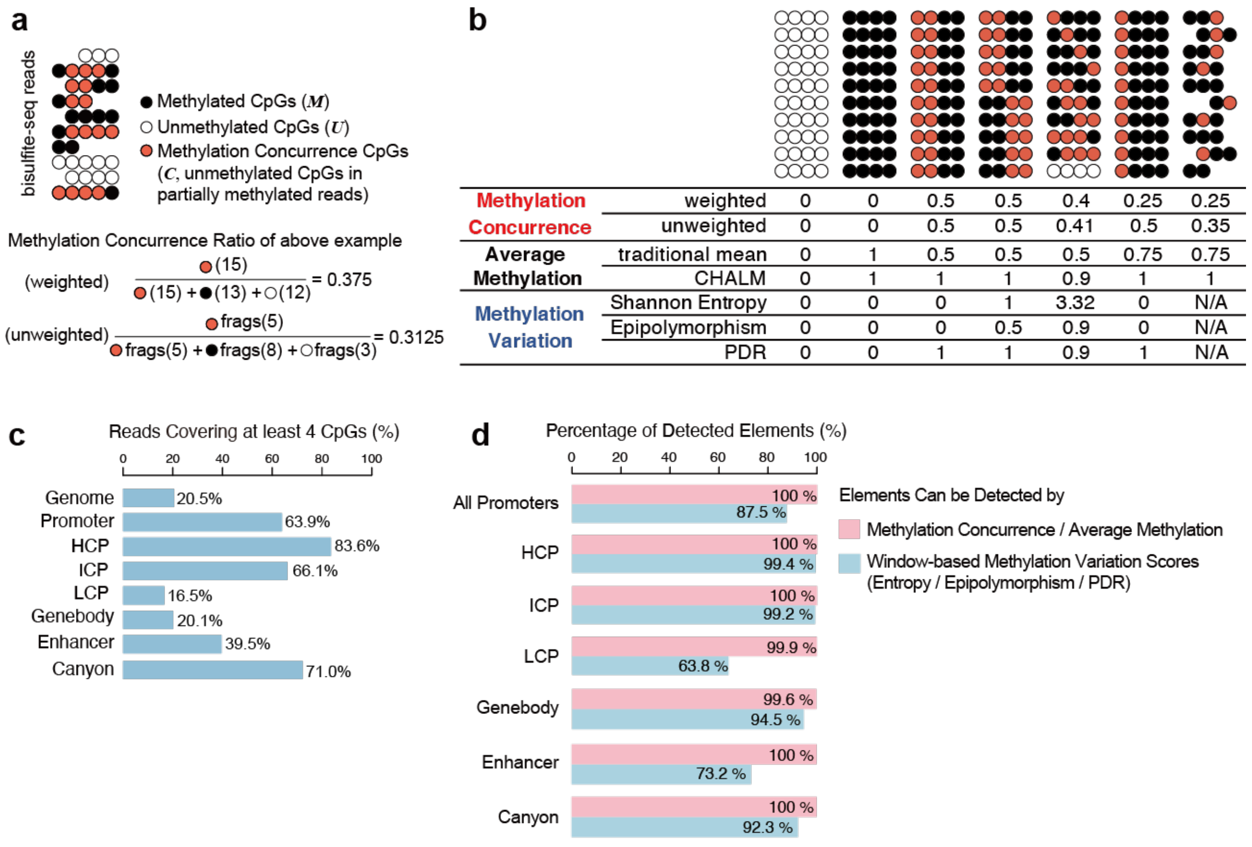

Supplementary Figure 1. The definition of methylation concurrence ratio, and its comparison with different methylation metrics. (a) The calculations of the weighted and unweighted versions of methylation concurrence. Solid circles are methylated cytosines. Blank circles are unmethylated cytosines. Red circles are unmethylated cytosines in partially methylated reads, i.e., methylation-concurrence cytosines. Equations below show the calculation of weighted and unweighted versions of methylation concurrence ratio using the example above. 'frags' is short for 'fragments'. **(b)** The calculations of different methylation metrics using examples of bisulfite-seq reads, which cover a 4-CpG region. As suggested in the previous studies, methylation Entropy, Epipolymorphism, and PDR only take reads covering at least 4 CpGs. Therefore, in the right-most example, the calculation of methylation variation is not available (indicated by N/A). **(c)** The percentage of reads covering at least 4 CpGs in the whole genome and different elements in CD3+ T-cells. HCP, high-CpG promoters; ICP, intermediate -CpG promoters; LCP, low-CpG promoters. **(d)** The percentage of different elements detected by methylation concurrence, average methylation, or methylation variation scores.

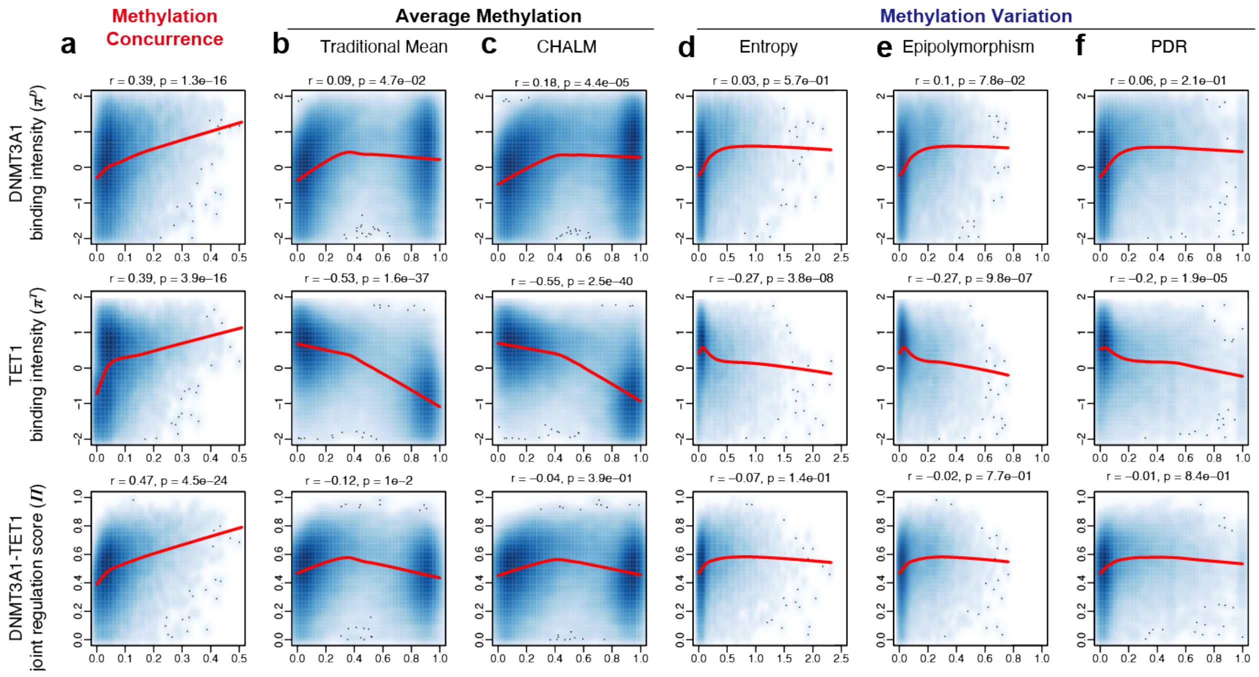

Supplementary Figure 2. Methylation concurrence ratio is positively correlated with both DNMT3A1 binding and TET1 binding in mouse embryonic stem cells (mESCs) (**a**) Correlation of methylation concurrence ratio versus DNMT3A1 binding (1st row) and TET1 binding (2nd row) in gene promoter regions. (**b**) Correlation of traditional mean methylation ratio versus DNMT3A1 binding (1st row) and TET1 binding (2nd row). (**c**) Correlation of CHALM versus DNMT3A1 binding (1st row), TET1 binding (2nd row), and 'DNMT3A1-TET1 joint regulation score' (3rd row) at promoters. (**d**) Correlation of methylation entropy versus DNMT3A1 binding (1st row) and TET1 binding (2nd row). (**e**) Correlation of Epipolymorphism versus DNMT3A1 binding (1st row), TET1 binding (2nd row), and 'DNMT3A1-TET1 joint regulation score' (3rd row) at promoters. (**f**) Correlation of PDR versus DNMT3A1 binding (1st row), TET1 binding (2nd row), and 'DNMT3A1-TET1 joint regulation score' (3rd row) at promoters. Spearman's rank correlation was calculated. P-values were calculated by the two-tailed correlation test for Spearman's correlation. LOWESS lines were plotted to describe the relationships between variables (indicated by red curves).

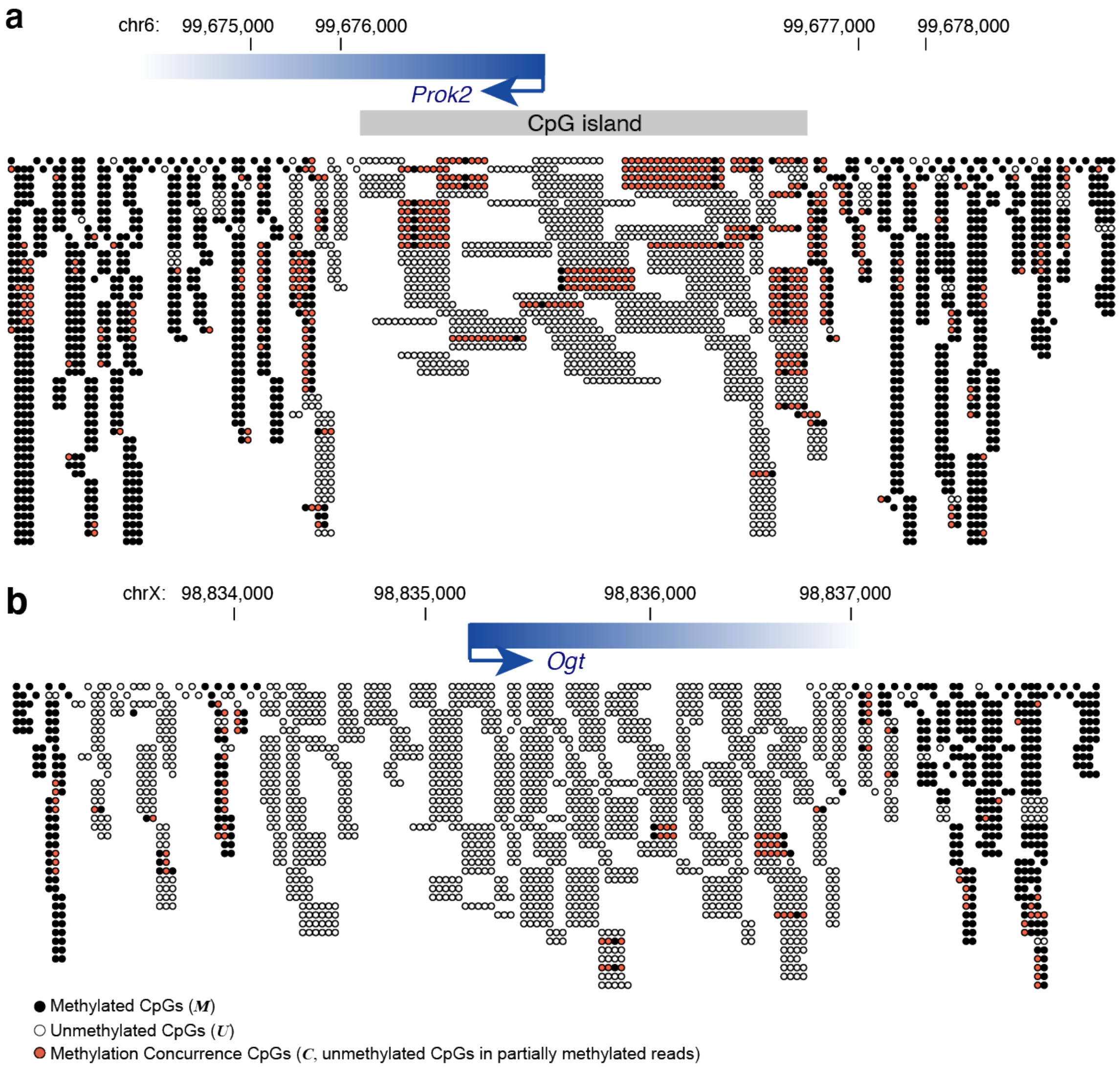

Supplementary Figure 3. The bisulfite-seq reads at the locus of genes *Prok2* and *Ogt* in mouse ESCs.
(a) Bisulfite-seq reads at *Prok2* gene promoter. (b) Same as (a), but for gene *Ogt*. Each consecutive horizontal sequence of circles represents a bisulfite-seq read. Solid circles are methylated cytosines. Blank circles are unmethylated cytosines. Red shaded circles are unmethylated cytosines in partially methylated reads, i.e., methylation-concurrence cytosines. CpG islands are shown in grey.

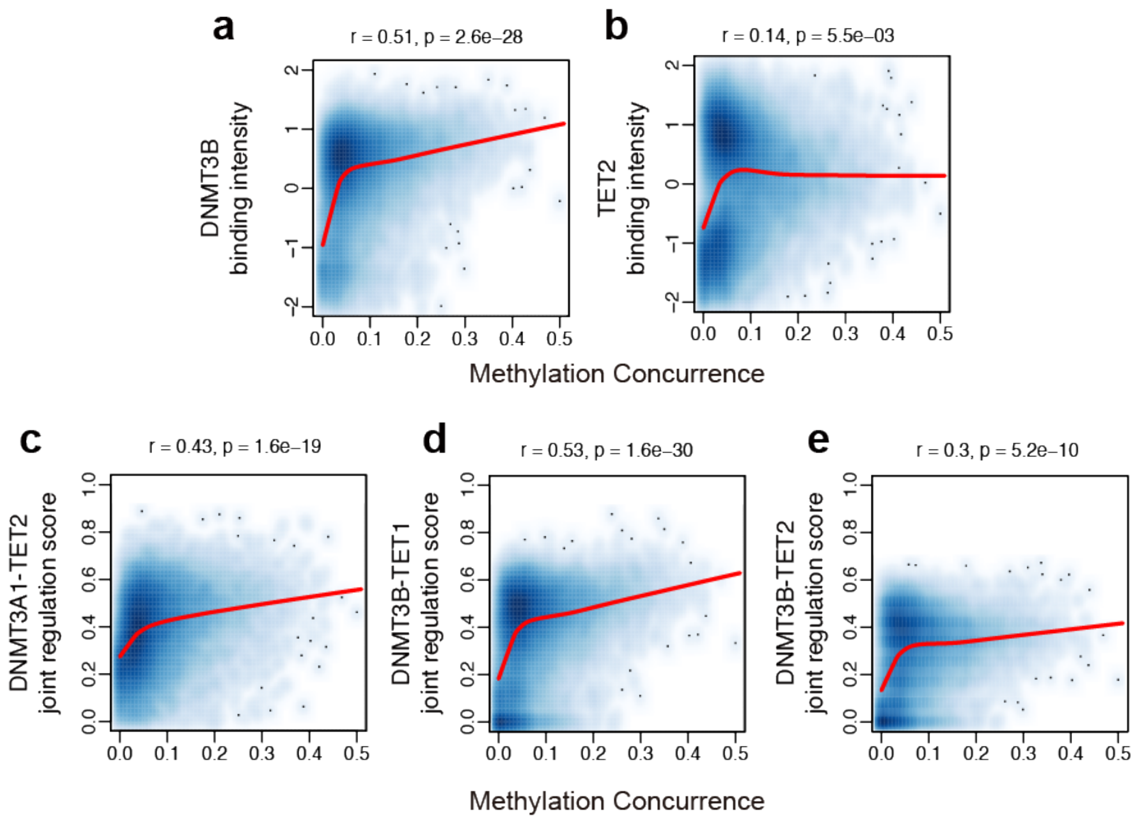

Supplementary Figure 4. The methylation concurrence can delineate the concurrence effect of other DNMT3 and TET enzymes in mouse ESCs. Binding intensities of both (a) DNMT3B and (b) TET2 are positively correlated with methylation concurrence at gene promoters. The (c) 'DNMT3A&TET2', (d) 'DNMT3B&TET1', and (e) 'DNMT3B&TET2' joint-regulation scores are positively correlated with methylation concurrence. Spearman's rank correlation was calculated. P-values were calculated by the two-tailed correlation test for Spearman's correlation. LOWESS lines were plotted to describe the relationships between variables (indicated by red curves).

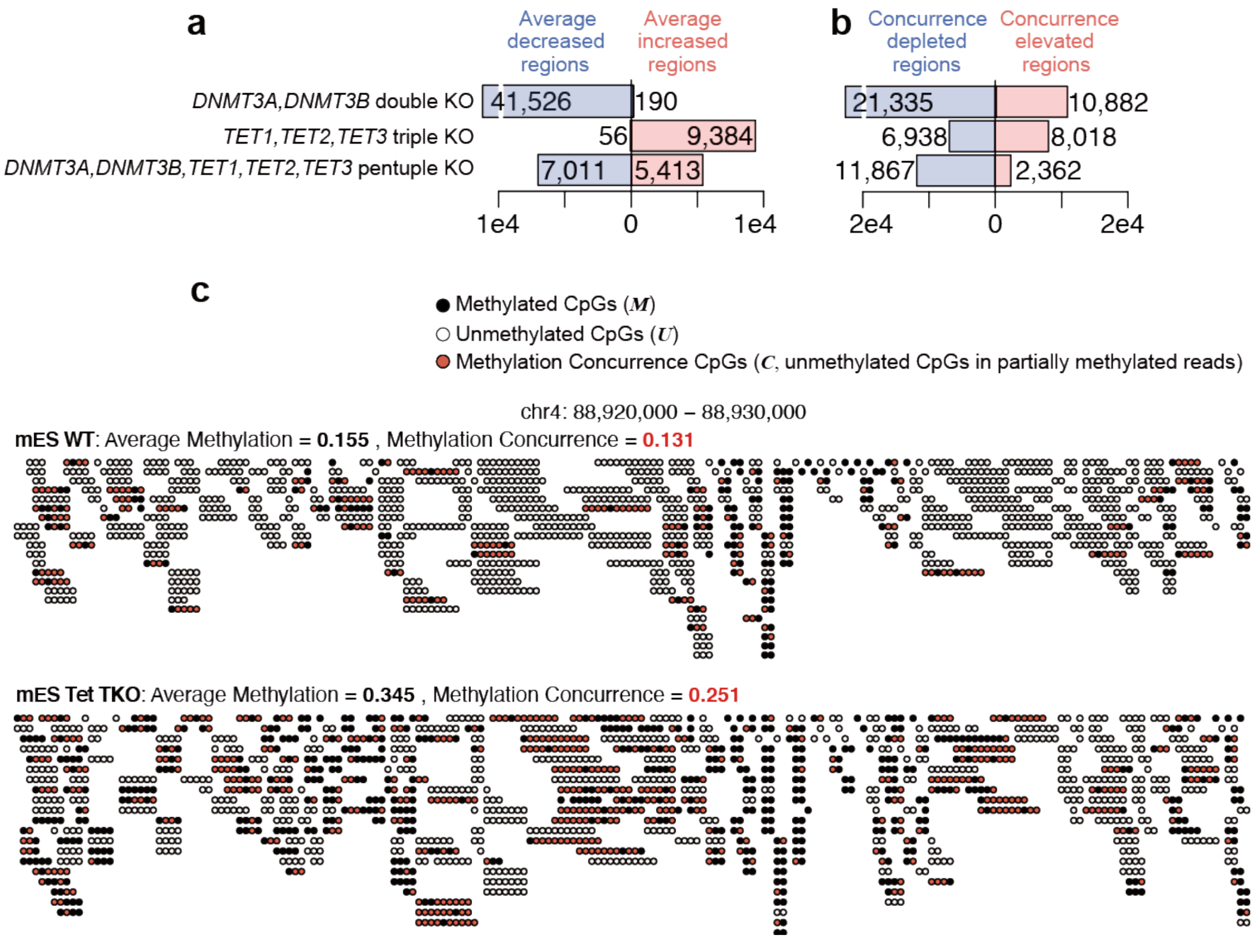

Supplementary Figure 5. DNA methylation alterations in *DNMT3* and/or *TET* knockout samples. (a) Average methylation altered regions in *DNMT3A* and *DNMT3B* double KO(DKO), *TET1, TET2, TET3* triple KO(TKO), and *DNMT3A, DNMT3B, TET1, TET2, TET3* pentuple KO(PKO) human embryonic stem cells (HUES8). (b) Methylation concurrence altered regions in DKO, TKO, and PKO human embryonic stem cells (HUES8). (c) Bisulfite-seq reads at an example region (chr4:88,920,000-88,930,000) show that, in some cases, methylation concurrence is elevated in *Tet* TKO mouse ESC.

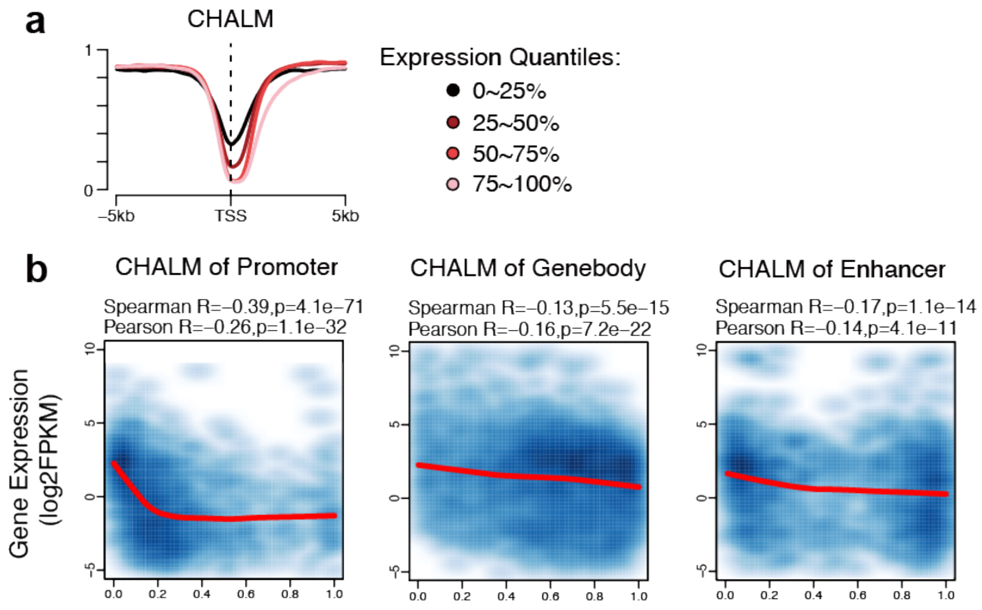

Supplementary Figure 6. The correlation between cellular heterogeneity-adjusted mean methylation (CHALM) and gene expression.
(**a**) Average profiles of CHALM in TSS proximal regions of four groups of genes in CD3+ T-cells. The four gene groups are the same as in Fig 2a. (**b**) The promoter CHALM scores (1st column) are negatively associated with gene expression level in CD3+ T-cells. Correlation between gene-body (2nd column) and enhancer (3rd column) CHALM scores and gene expression are lower than at the promoter. Promoter regions are from 1kb upstream to 500bp downstream of TSS. Gene-body regions are from 500bp downstream of TSS to TTS. Enhancer regions are defined based on chromatin interactions validated by Hi-C data (see Methods). Spearman's rank correlation and Pearson's correlation were calculated. P-values were calculated by the two-tailed correlation test. LOWESS lines were plotted to describe the relationships between variables (indicated by red curves).

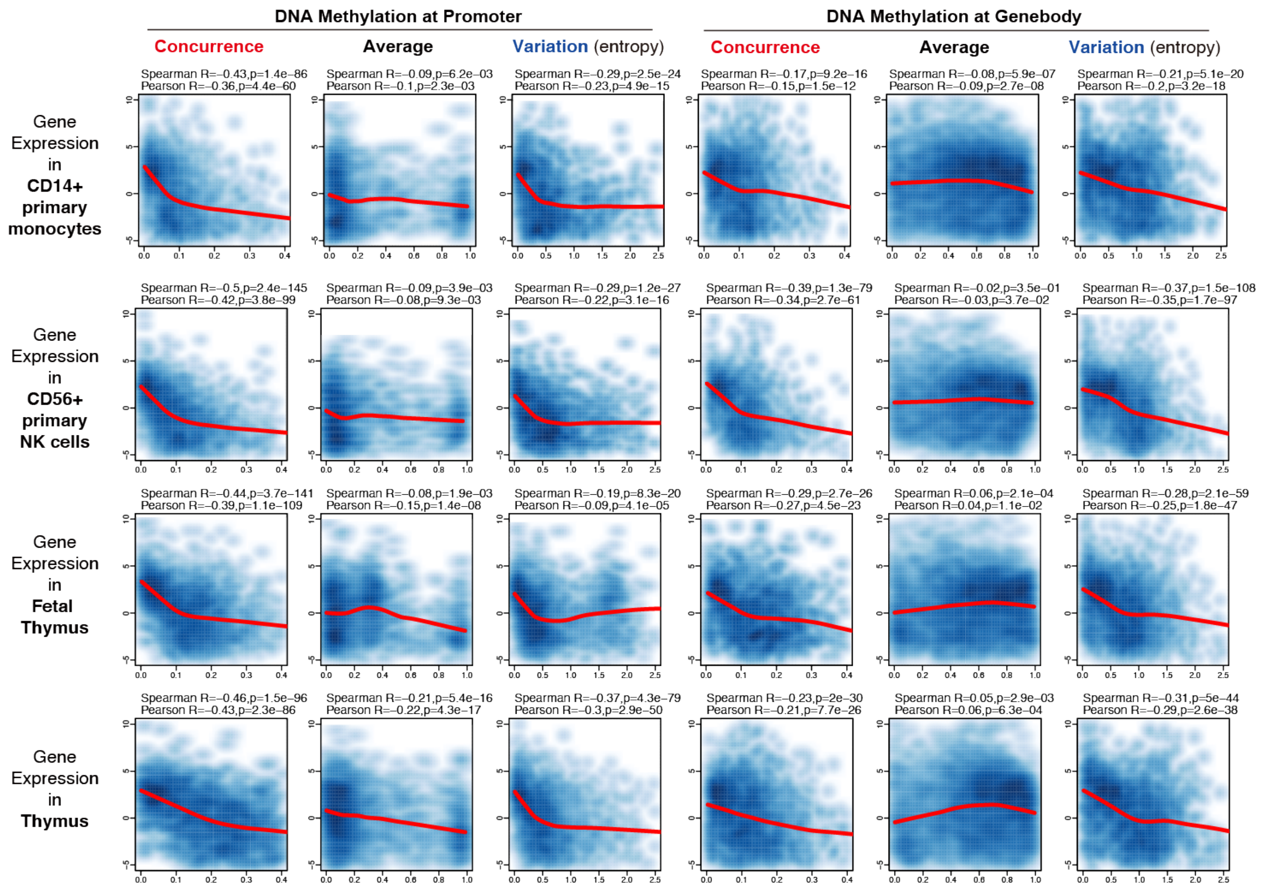

Supplementary Figure 7. The methylation concurrence ratio is consistently associated with gene expression in various samples.

The Spearman correlation between gene expression and promoter methylation concurrence (1st column), promoter average methylation (2nd column), gene-body methylation concurrence (3rd column), gene-body average methylation (4th column) in 4 different samples, i.e., CD14+ primary cells (1st row), CD56+ primary cells (2nd row), fetal thymus tissue (3rd row), and adult thymus tissue (4th row). Spearman's rank correlation and Pearson's correlation were calculated. P-values were calculated by the two-tailed correlation test. LOWESS lines were plotted to describe the relationships between variables (indicated by red curves). To increase reliability, we select regulatory elements whose CpGs are all sufficiently covered (⩾4 reads). To make fair comparisons, only the elements which can be detected by all three metrics are included. This results in: 9,817 promoter regions and 14,678 gene-body regions in CD14+ primary cells; 10,173 promoter regions and 15,547 gene-body regions in CD56+ primary cells; 10,957 promoter regions and 16,914 gene-body regions in fetal thymus; 11,509 promoter regions and 18,914 gene-body regions in adult thymus.

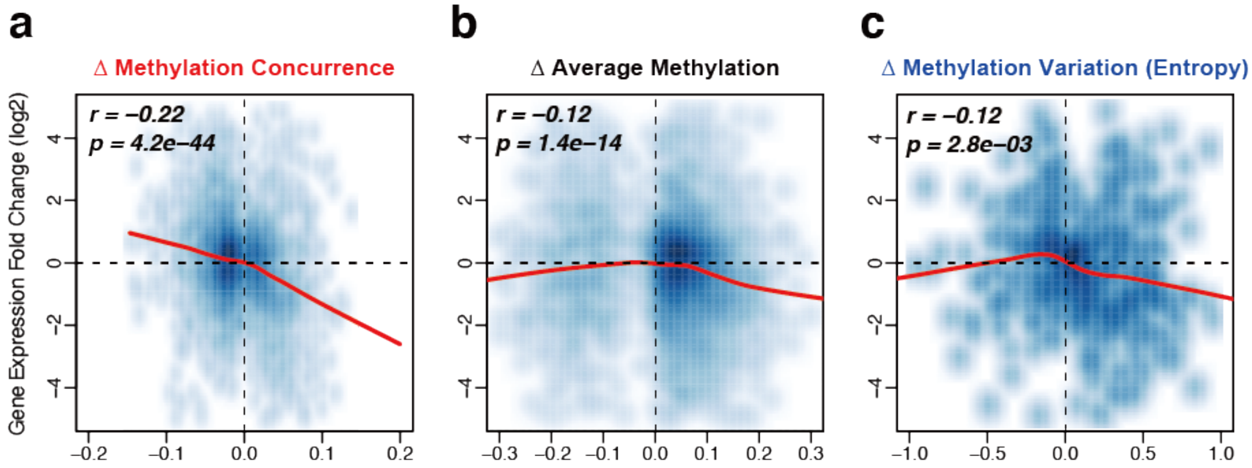

Supplementary Figure 8. The correlation between gene expression change and promoter methylation difference

Scatter plots showing the correlation between gene expression changes and the differences (Δ) of methylation concurrence (left panel), average methylation (middle panel), and methylation variation (right panel) between lung cancer and normal lung samples. All genes' promoters are included for analysis, but only those exhibiting a significant methylation change between cancer and normal are plotted. This results in 3,759 promoters for methylation concurrence, 3,956 promoters for average methylation, and 575 promoters for methylation variation. Pearson's correlation was used. P-values were calculated by the two-tailed correlation test. LOWESS lines were plotted to describe the relationships between variables (indicated by red curves).

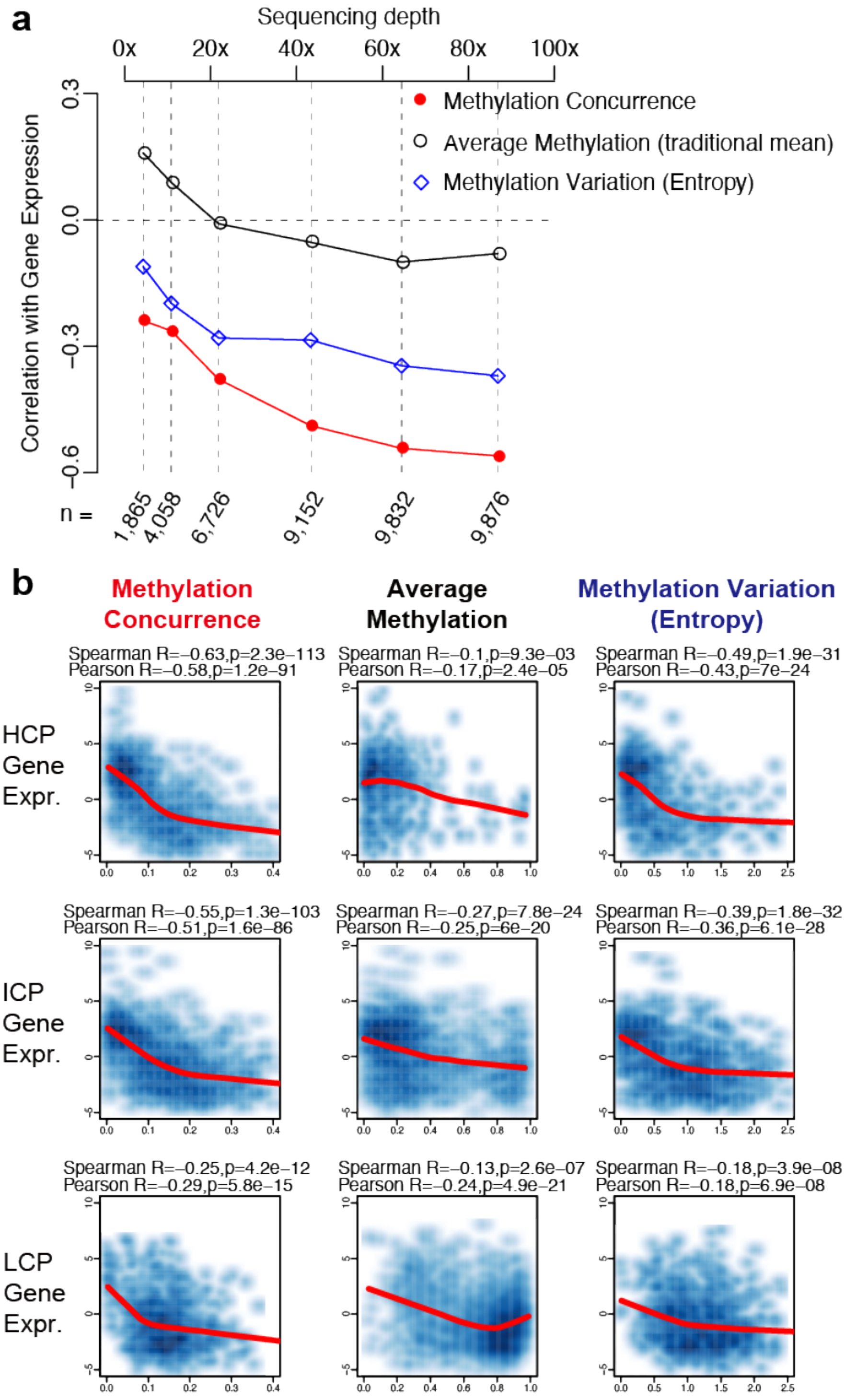

Supplementary Figure 9. Effects of sequencing depth and CpG density on quantification of methylation concurrence

(**a**) Spearman correlations between methylation quantitative measures and gene expression at varying sequencing depths. Y-axis indicates the Spearman correlation between gene expression and three methylation quantitative measures of gene promoters in CD3+ T-cells. The X-axis shows the average sequencing depth of down-

sampled bisulfite-seq data. The numbers of included promoters are indicated below. (**b**) Scatter plots show the correlation of HCP (1st row, n=6,679), ICP (2nd row, n=5,604), and LCP (3rd row, n=2,117) gene expression versus promoter methylation concurrence (1st column), average methylation (2nd column), and methylation variation (entropy, 3rd column), in CD3+ T-cells. Spearman's rank correlation and Pearson's correlation were calculated. P-values were calculated by the two-tailed correlation test.

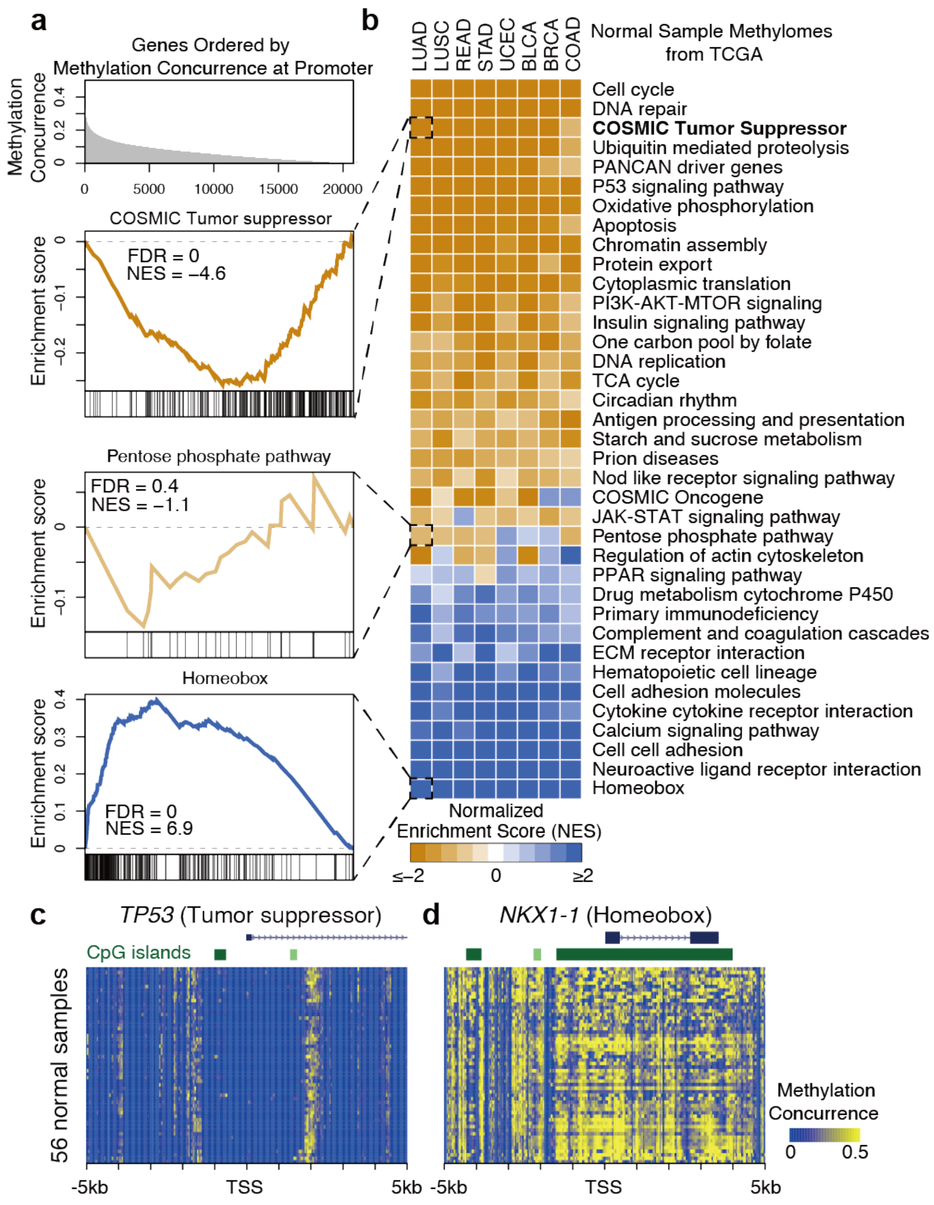

Supplementary Figure 10. Tumor suppressor genes are characterized by low methylation concurrence level in normal tissues

(**a**) Genes were decreasingly ordered by promoter methylation concurrence ratios (1st row). GSEA running enrichment scores of ‘COSMIC Tumor suppressor’ (2nd row), ‘Pentose phosphate pathway’ (3rd row), and ‘Homeobox’ genes (4th row) in TCGA-LUAD normal sample are shown. Normalized enrichment scores (NES) and FDR values were given by GSEA software. (**b**) Heatmap showing GSEA NES in TCGA normal samples related to 8 cancer types. Pathways and functional terms are curated by GSEA MutSigDB, except ‘COSMIC Tumor suppressor’, ‘COSMIC Oncogene’, and ‘PANCAN driver genes’ (see Methods). (**c**) Methylation concurrence profiles in TSS proximal regions of tumor suppressor *TP53* in 56 normal human methylomes. (**d**) Same as (c), but for Homeobox gene *NKX1-1*.

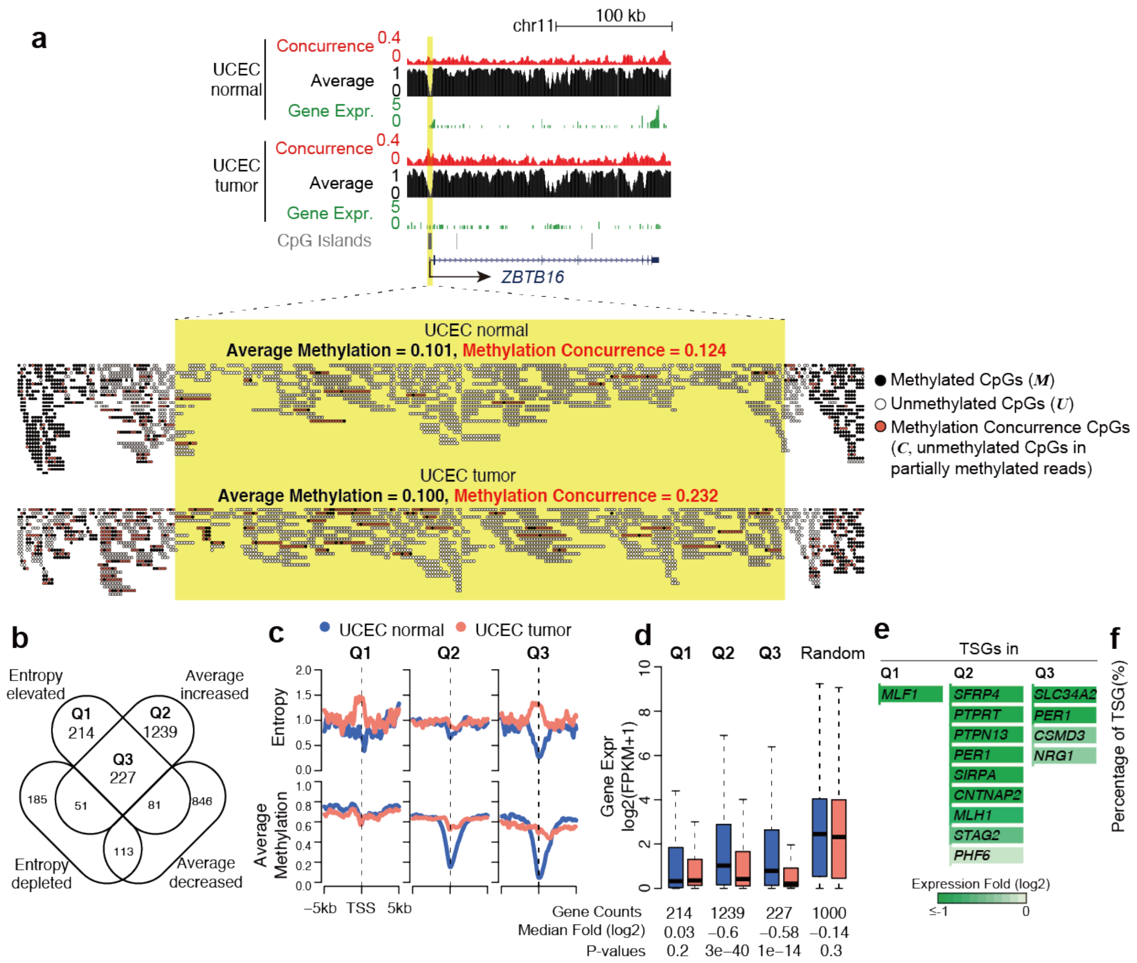

Supplementary Figure 11. (**a**) The methylation concurrence, average methylation, and RNA-seq reads densities around *ZBTB16* gene locus in UCEC normal and tumor samples. Bisulfite-seq reads at the *ZBTB16* gene promoter region (yellow shaded) are shown below. (**b**) Venn diagram shows the overlap between promoter methylation entropy altered genes and average methylation altered genes in the TCGA uterus tumor (UCEC). Q1, entropy-elevated but average-methylation stable genes; Q2, hypermethylated but entropy-stable genes; Q3, entropy-elevated and hypermethylated genes. (**c**) Average profiles of methylation entropy (upper panel) and average methylation (lower panel) in TSS regions of Q1, Q2, and Q3 genes. UCEC normal is in blue, and UCEC tumor is in red. (**d**) Gene expression change of Q1, Q2, Q3, and 1,000 randomly selected genes. The fold changes between median values (log2 scale) are indicated below. A two-tailed Wilcoxon signed-rank test was used for the significance test. The line in the box center refers to the median, the limits of box refer to the 25th and 75th percentiles and whiskers are plotted at the highest and lowest points within the 1.5 times interquartile range. (**e**) COSMIC tumor suppressor genes which are overlapped with Q1, Q2, and Q3 genes. Gene expression fold changes (log2 scale) are indicated. Darker green means higher repression in the UCEC tumor sample. (**f**) The percentages of tumor suppressors in Q1, Q2, and Q3 genes.

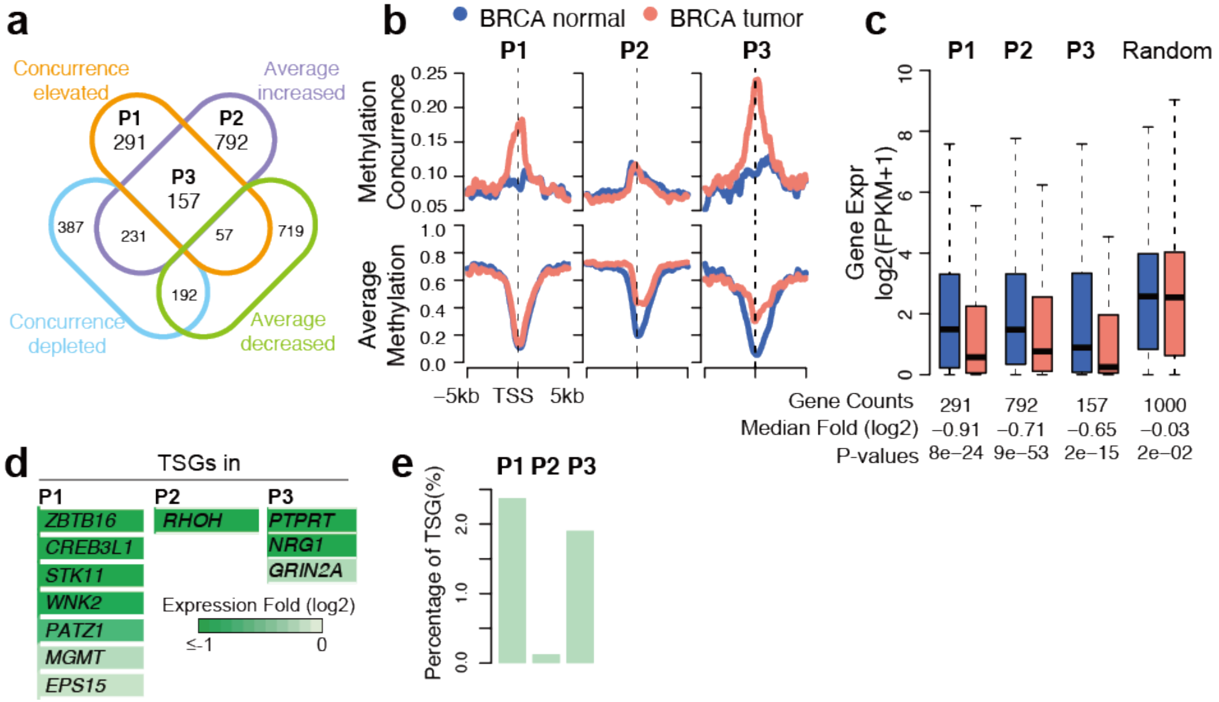

Supplementary Figure 12. Cancer-related genes can be repressed by methylation concurrence elevation rather than hypermethylation in breast tumor

(**a**) Venn diagram shows the overlap between promoter methylation concurrence altered genes and average methylation altered genes in TCGA breast tumor (BRCA). P1, concurrence-elevated but average-methylation-stable genes; P2, hypermethylated but concurrence-stable genes; P3, concurrence-elevated and hypermethylated genes. (**b**) Average profiles of methylation concurrence (upper panel) and average methylation (lower panel) in TSS regions of P1, P2, and P3 genes. The BRCA normal is shown in blue, while the BRCA tumor is shown in red. (**c**) Gene expression change of P1, P2, P3, and 1,000 randomly selected genes. The fold changes between median values (log2 scale) are indicated below. A two-tailed Wilcoxon signed-rank test was used for the significance test. The line in the box center refers to the median, the limits of box refer to the 25th and 75th percentiles and whiskers are plotted at the highest and lowest points within the 1.5 times interquartile range. (**d**) COSMIC tumor suppressors which are overlapped with P1, P2, and P3 genes. Gene expression fold changes (log2 scale) are indicated. Darker green signifies higher repression in the BRCA tumor sample. (**e**) The percentages of tumor suppressors in P1, P2, and P3 genes.

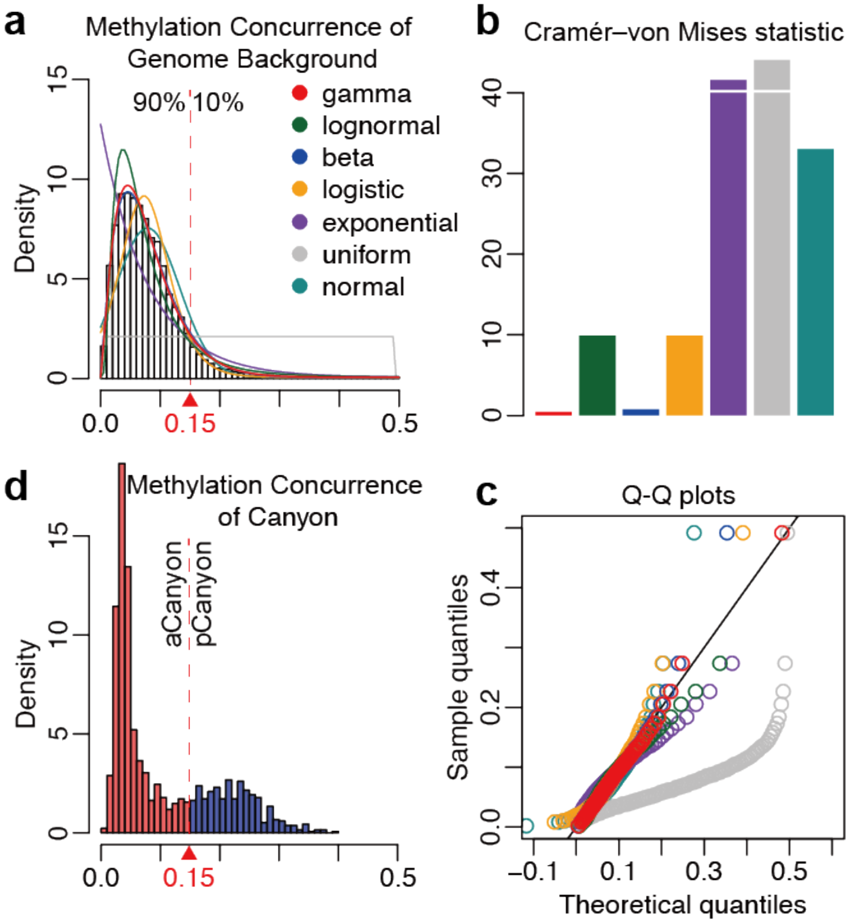

Supplementary Figure 13. Determining the threshold of methylation concurrence ratio for categorizing methylation canyons

(**a**) Methylation concurrence ratios of genome background in CD3+ T-cells are fitted to various distribution models, including 'gamma', 'log-normal', 'beta', 'normal', 'uniform', 'exponential', and 'logistic' distributions. The curves indicate the fitted models based on parameters from maximum likelihood estimation. (**b**) Judging the goodness of fitness in (a) using the Cramér–von Mises criterion. (**c**) Q-Q plots comparing the sample distribution with theoretical distributions. (**d**) Methylation canyons are categorized by cutoff from the genome background (indicated as the red triangle and vertical dashed lines).

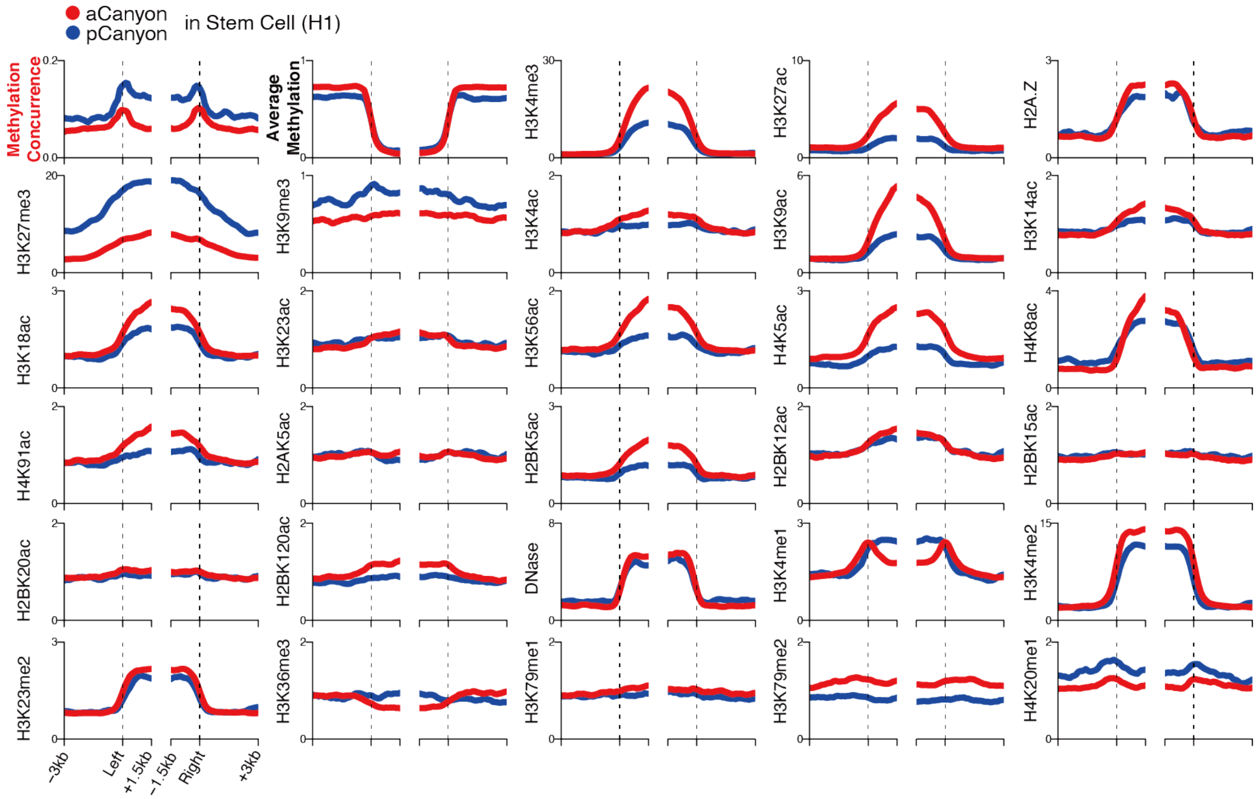

Supplementary Figure 14. Average profiles of methylation concurrence, average methylation, and 28 histone modifications/variants in methylation canyons in H1 stem cells. 'aCanyons' (red) are low-concurrence canyons. 'pCanyons' (blue) are high-concurrence canyons. The X-axis indicates the distance to canyon borders.

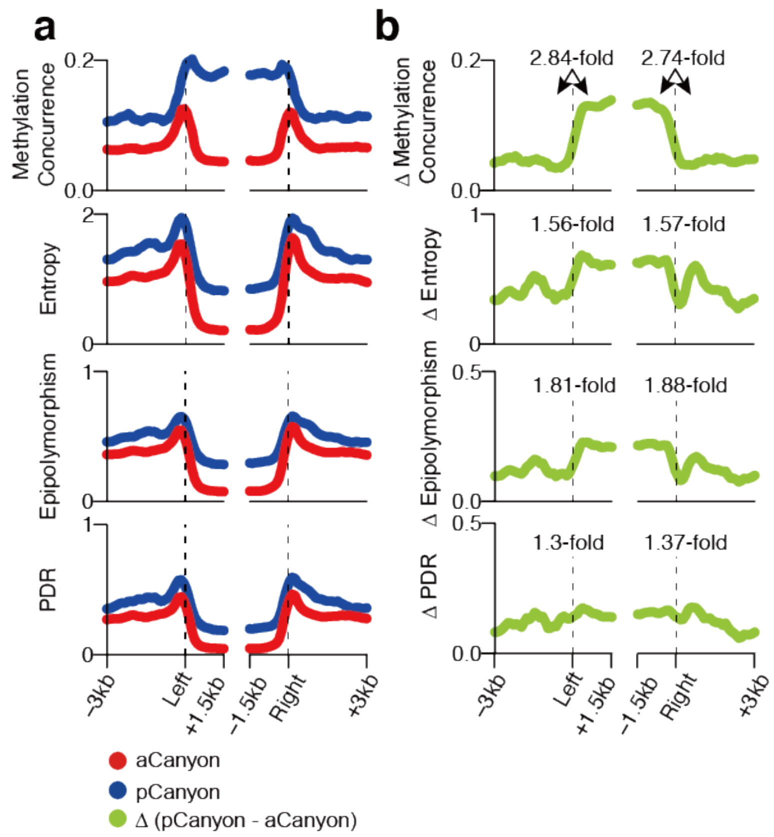

Supplementary Figure 15. The profiles of methylation concurrence and methylation variation scores at two canyon groups

(**a**) Average profiles of methylation concurrence, methylation Entropy, Epipolymorphism, and PDR on methylation canyons in CD3+ T-cells. 'aCanyons' (red) are low-concurrence canyons. 'pCanyons' (blue) are high-concurrence canyons. The X-axis indicates the distance to canyon borders. (**b**) The difference (Δ) between pCanyons and aCanyons for scores in (a).

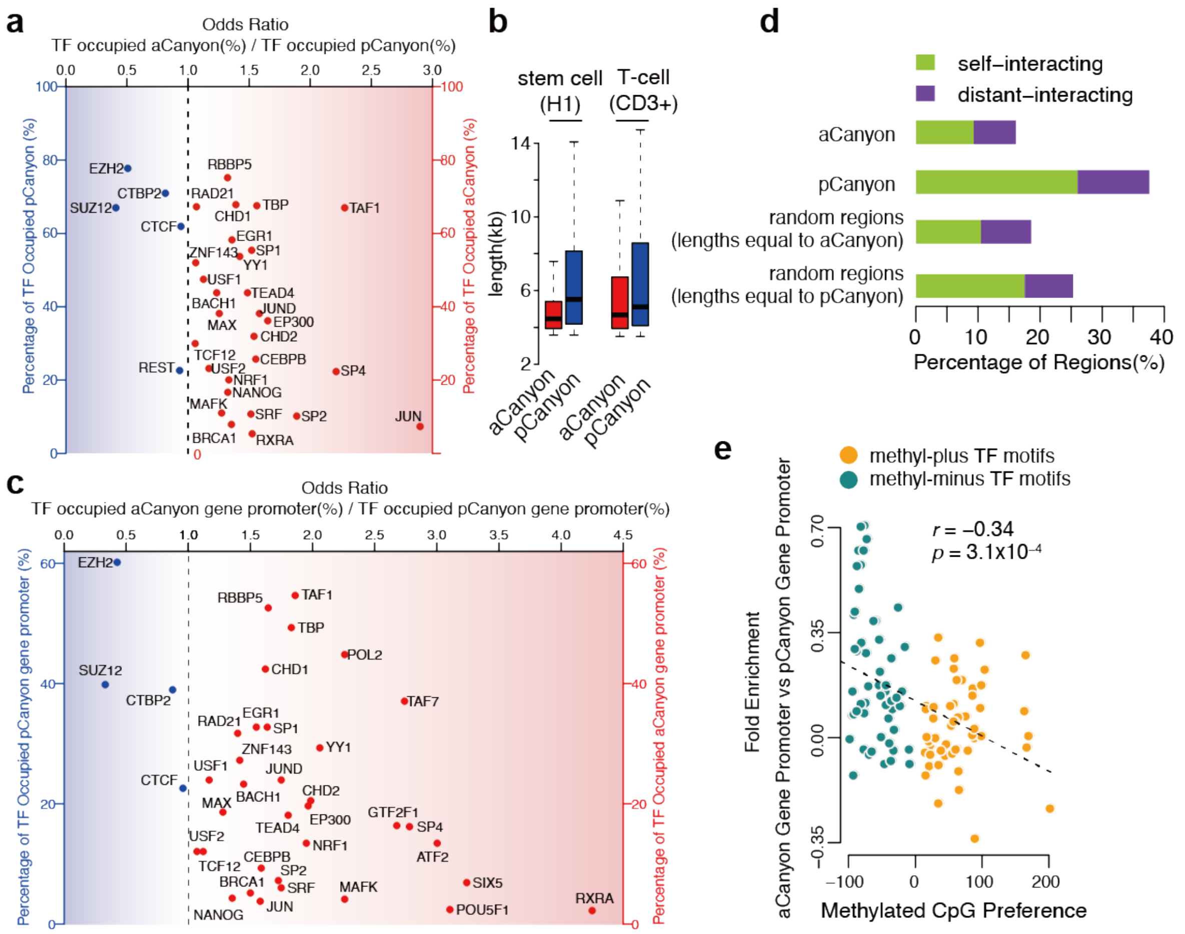

Supplementary Figure 16. The differences of transcription factor binding and chromatin interactions between canyon groups

(**a**) Scatter plot showing TF binding difference between aCanyon and pCanyon in H1 human stem cells. X-axis indicates the odds ratio between TF-bound-aCanyon (%) and TF-bound-pCanyon (%). For the left portion (blue shaded), the Y-axis is the percentage of pCanyons bound by TF. For the right portion (red shaded), the Y-axis is the percentage of aCanyons bound by TF. Each dot represents a TF. (**b**) The length of aCanyons and pCanyons in stem cells (H1) and CD3+ T-cells. The line in the box center refers to the median, the limits of box refer to the 25th and 75th percentiles and whiskers are plotted at the highest and lowest points within the 1.5 times interquartile range. (**c**) Scatter plot showing TF binding difference between aCanyon gene promoters and pCanyon gene promoters in H1 human stem cells. X-axis indicates the odds ratio between TF-bound-aCanyon-gene-promoter (%) and TF-bound-pCanyon-gene-promoter (%). For the left portion (blue shaded), the Y-axis is the percentage of pCanyon gene promoters bound by TF. For the right portion (red shaded), the Y-axis is the percentage of aCanyon gene promoters bound by TF. (**d**) The chromatin interactions on methylation canyons. Percentages of canyons and random genomic regions that have high-order chromatin interactions in CD3+ T-cells. Chromatin interactions were defined by pairs of Hi-C anchors. 'Self-interacting' indicates the anchor pairs located in the same canyon/random region. 'Distant-interacting' indicates anchor pairs in which only one of the pair is in the

canyon/random region. (**e**) Relationship between fold enrichment at Canyon gene promoters and 5mC preference of TF motifs. Each dot represents a motif. Y-axis indicates the fold change (log2) between enrichment at aCanyon gene promoters and enrichment at pCanyon gene promoters of the same motif. The X-axis shows the 5mC preference of motifs measured by the SELEX technique. ‘methyl-plus’ TFs prefer to bind methylated sequences, while binding of ‘methyl-minus’ TFs are not favored by 5mC. Spearman’s rank correlation was used. P-values were calculated by the two-tailed correlation test for Spearman’s correlation. The linear model was plotted to describe the relationships between variables (indicated by the dashed line).

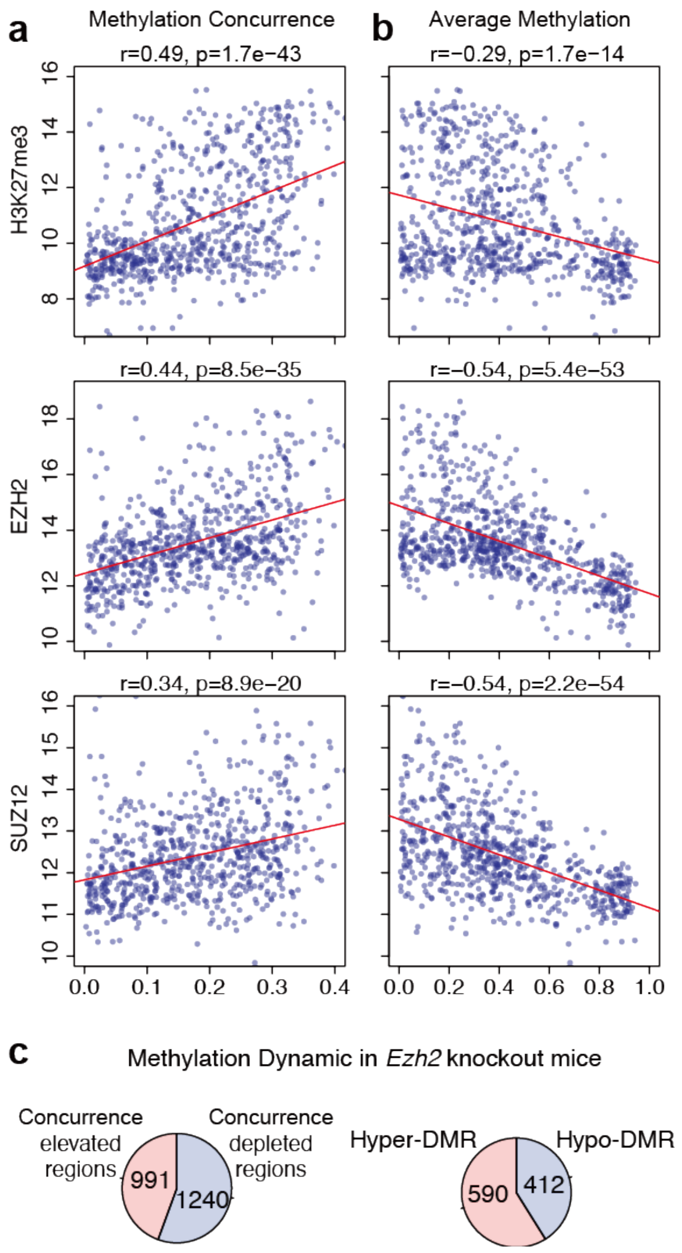

Supplementary Figure 17. Methylation concurrence is promoted by Polycomb complex binding

(**a**) Methylation concurrence is positively correlated with H3K27me3, EZH2, and SUZ12 binding in promoter regions in H1 human stem cells. (**b**) Average methylation is negatively associated with Polycomb binding in H1. Spearman's rank correlation was used in (a) and (b). P-values were calculated by the two-tailed correlation test for Spearman's correlation. Linear models were plotted to describe the relationships between variables (indicated by red lines). (**c**) *Ezh2* knockout in mice leads to more methylation-concurrence depleted regions (left) and more hypermethylated DMRs (right).

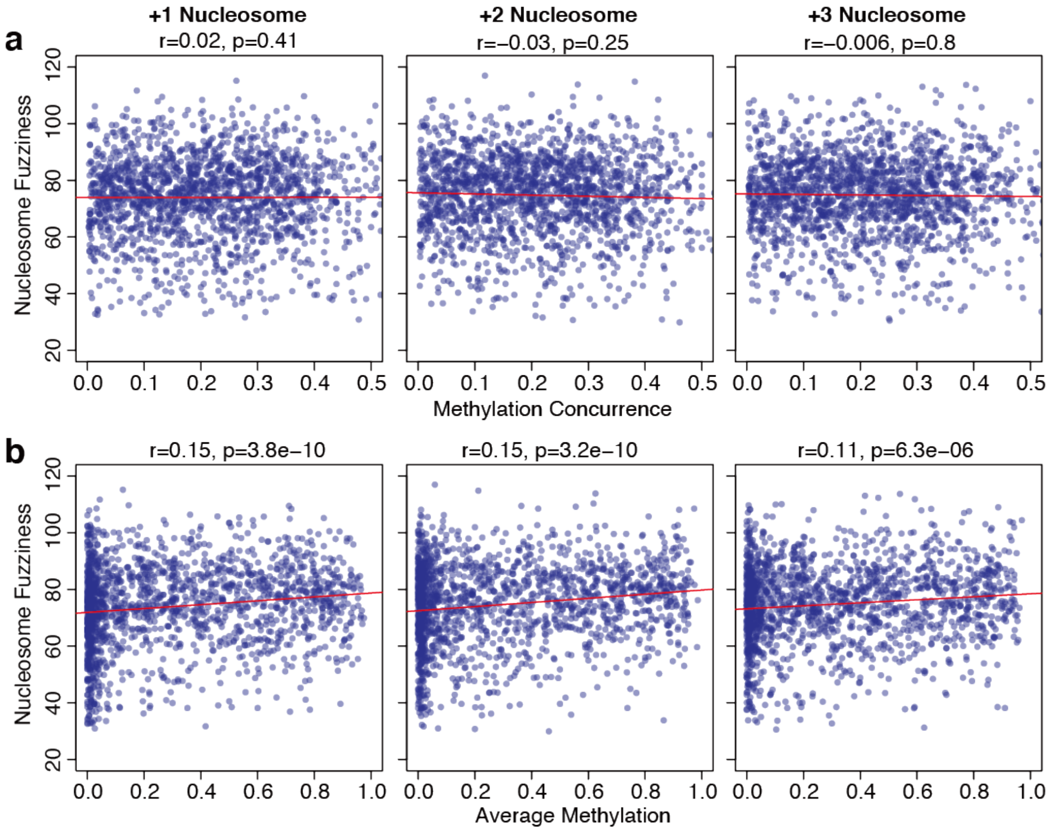

Supplementary Figure 18. Methylation concurrence is independent of nucleosome positioning fuzziness

(**a**) The methylation concurrence ratios of +1, +2, +3 nucleosomes are not correlated with fuzziness in human brain tissue. (**b**) A weak correlation is observed between average methylation and nucleosome fuzziness in human brain tissue. Spearman's rank correlation was used in (a) and (b). P-values were calculated by the two-tailed correlation test for Spearman's correlation. Linear models were plotted to describe the relationships between variables (indicated by red lines).

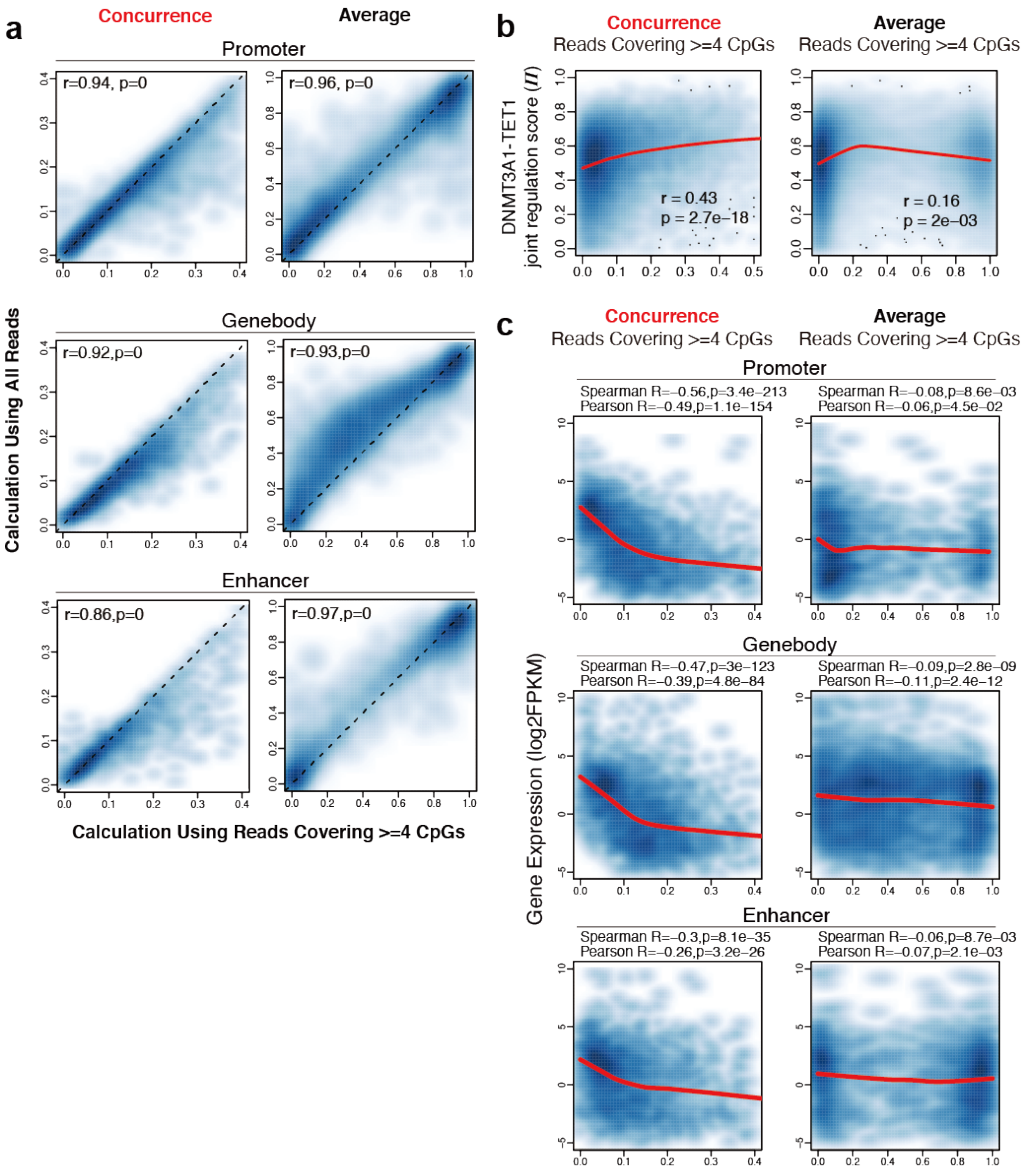

Supplementary Figure 19. The calculations of methylation concurrence and average methylation only using reads covering at least 4 CpGs.
(a) In CD3+ T-cells, the calculations of methylation concurrence (left) and average methylation (right) only using reads covering >= 4 CpGs (X-axis) are highly correlated with the calculations using all reads (Y-axis). Pearson's correlation was calculated. P-values were calculated by the two-tailed correlation test for Pearson's correlation. The diagonal line (slashed) indicates Y equal to X. **(b)** In mouse ESCs, the promoter

methylation concurrence calculated using reads covering >= 4 CpGs significantly better correlates with 'DNMT3A1-TET1 joint regulation score' than average methylation does. Spearman's rank correlation was calculated. P-values were calculated by the two-tailed correlation test for Spearman's correlation. **(c)** The promoter/gene-body/enhancer methylation concurrence calculated using reads covering >= 4 CpGs are strongly negatively correlated with gene expression level in CD3+ T-cells, and this correlation is stronger than that of the average methylation. Spearman's rank correlation and Pearson's correlation were calculated based on all data points. P-values were calculated by the two-tailed correlation test. LOWESS lines were plotted to describe the relationships between variables (indicated by red curves).

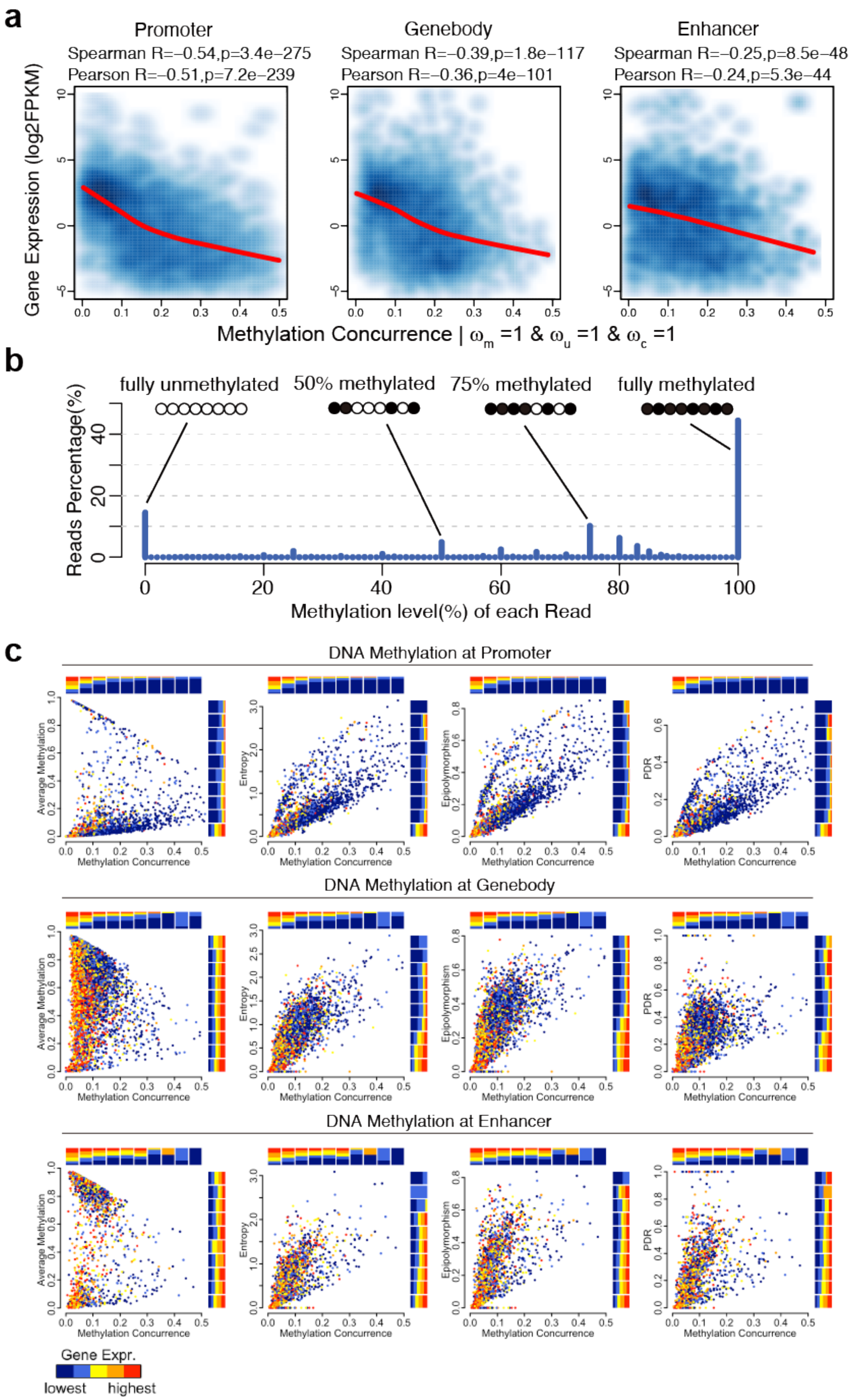
a
Promoter
Spearman R=−0.54,p=3.4e−275
Pearson R=−0.51,p=7.2e−239
Genebody
Spearman R=−0.39,p=1.8e−117
Pearson R=−0.36,p=4e−101
Enhancer
Spearman R=−0.25,p=8.5e−48
Pearson R=−0.24,p=5.3e−44
Gene Expression (log2FPKM)
Methylation Concurrence | ωm =1 & ωu =1 & ωc =1
b
fully unmethylated
50% methylated
75% methylated
fully methylated
Reads Percentage(%)
Methylation level(%) of each Read
c
DNA Methylation at Promoter
DNA Methylation at Genebody
DNA Methylation at Enhancer
Average Methylation
Entropy
Epipolymorphism
PDR
Methylation Concurrence
Gene Expr.
lowest
highest

Supplementary Figure 20. (**a**) Correlations between unweighted methylation concurrence ratios and gene expression. The methylation concurrence ratios in CD3+ T-cells were calculated with $\omega_m = 1$, $\omega_u = 1$, and $\omega_c = 1$. Scatter plots show the correlation between gene expression and methylation concurrence ratios on the promoter (left), gene-body (middle), and enhancer (right). Spearman's rank correlation and Pearson's correlation are calculated. P-values were calculated by the two-tailed correlation test. LOWESS lines were plotted to describe the relationships between variables (indicated by red curves). (**b**) Histogram of the methylation level of each bisulfite-seq read from CD3+ T-cells. (**c**) The scatter plots compare methylation concurrence and other methylation metrics in terms of gene expression correlation in CD3+ T-cells. For each panel, the methylation concurrence ratios are on the X-axis. Other methylation metrics (i.e., average methylation, Entropy, Epipolymorphism, PDR) are on the Y-axis. Colors indicate the gene expression quantiles.
