# Supplementary material for: The concurrence of DNA methylation and demethylation is associated with transcription regulation
Source: Nat Commun. 2021 Sep 6;12:5285. doi: 10.1038/s41467-021-25521-7 (PMC8421433; doi:10.1038/s41467-021-25521-7)
Supplement: Supplementary file 3 — Description of Additional Supplementary Files [file 41467_2021_25521_MOESM3_ESM.pdf]

Description of Additional Supplementary Files

File Name: Supplementary Data 1
Description: Datasets used in this study

File Name: Supplementary Data 2
Description: Lists of genes repressed by promoter methylation concurrence-elevation, hypermethylation, or both of them in uterus tumor

File Name: Supplementary Data 3
Description: List of methylation canyons and their target genes in CD3+ T-cells

File Name: Supplementary Data 4
Description: List of ‘methyl-plus’ and ‘methyl-minus’ motifs used in this study. Enrichment at aCanyon and pCanyon in CD3+ T-cells is indicated.
