# Supplementary material for: The concurrence of DNA methylation and demethylation is associated with transcription regulation
Source: Nat Commun. 2021 Sep 6;12:5285. doi: 10.1038/s41467-021-25521-7 (PMC8421433; doi:10.1038/s41467-021-25521-7)
Supplement: Supplementary file 8 — Reporting Summary [file 41467_2021_25521_MOESM8_ESM.pdf]

nature research

Corresponding author(s): Wei Li

Last updated by author(s): Jul 16, 2021

Reporting Summary

Nature Research wishes to improve the reproducibility of the work that we publish. This form provides structure for consistency and transparency in reporting. For further information on Nature Research policies, see our Editorial Policies and the Editorial Policy Checklist.

Statistics

For all statistical analyses, confirm that the following items are present in the figure legend, table legend, main text, or Methods section.

n/a	Confirmed	
☐	☒	The exact sample size (*n*) for each experimental group/condition, given as a discrete number and unit of measurement
☐	☒	A statement on whether measurements were taken from distinct samples or whether the same sample was measured repeatedly
☐	☒	The statistical test(s) used AND whether they are one- or two-sided<br>*Only common tests should be described solely by name; describe more complex techniques in the Methods section.*
☐	☒	A description of all covariates tested
☐	☒	A description of any assumptions or corrections, such as tests of normality and adjustment for multiple comparisons
☐	☒	A full description of the statistical parameters including central tendency (e.g. means) or other basic estimates (e.g. regression coefficient) AND variation (e.g. standard deviation) or associated estimates of uncertainty (e.g. confidence intervals)
☐	☒	For null hypothesis testing, the test statistic (e.g. *F*, *t*, *r*) with confidence intervals, effect sizes, degrees of freedom and *P* value noted<br>*Give P values as exact values whenever suitable.*
☐	☒	For Bayesian analysis, information on the choice of priors and Markov chain Monte Carlo settings
☐	☒	For hierarchical and complex designs, identification of the appropriate level for tests and full reporting of outcomes
☐	☒	Estimates of effect sizes (e.g. Cohen's *d*, Pearson's *r*), indicating how they were calculated

Our web collection on statistics for biologists contains articles on many of the points above.

Software and code

Policy information about availability of computer code

Data collection	No software was used for data collection
Data analysis	FastQC v0.11.7; Trim Galore v0.6.4; BSMAP v2.90; STAR v2.6.0c; RSEM v1.3.1; bowtie2 v2.2.7; DANPOS v2.2.2; Homer v4.8; samtools v0.1.19; Metilene v0.2-8; GSEA v4.0.3; bedtools v2.25.0; Methylation Concurrence (https://github.com/JiejunShi/CAMDA); DNA methylation heterogeneity scores(https://github.com/MPIIComputationalEpigenetics/WSHScripts); Replot GSEA (https://github.com/PeeperLab/Rtoolbox/blob/master/R/ReplotGSEA.R); DAVID online tool v6.8 (https://david.ncifcrf.gov/); Table Browser tool in the UCSC Genome Browser (http://genome.ucsc.edu/cgi-bin/hgTables); wigToBigWig from UCSC binary utility directory (http://hgdownload.soe.ucsc.edu/admin/exe/) .

For manuscripts utilizing custom algorithms or software that are central to the research but not yet described in published literature, software must be made available to editors and reviewers. We strongly encourage code deposition in a community repository (e.g. GitHub). See the Nature Research guidelines for submitting code & software for further information.

Data

Policy information about availability of data

All manuscripts must include a data availability statement. This statement should provide the following information, where applicable:

- Accession codes, unique identifiers, or web links for publicly available datasets
- A list of figures that have associated raw data
- A description of any restrictions on data availability

All the public or controlled data used by this study are summarized in Supplementary Data 1. In total, 75 methylomes (WGBS and reduced representation bisulfite sequencing (RRBS)) were collected from the Roadmap Epigenomics project (http://www.roadmapepigenomics.org/), Encyclopedia of DNA Elements (ENCODE) project (https://www.encodeproject.org/), The Cancer Genome Atlas (TCGA) project (https://portal.gdc.cancer.gov/), Gene Expression Omnibus (GEO, https://www.ncbi.nlm.nih.gov/geo/) and DNA Data Bank of Japan (DDBJ, https://www.ddbj.nig.ac.jp) databases, including 69 human and 6 mouse datasets. All of them are

publicly available except two matched normal-tumor pairs from TCGA, which need to apply for accession from GDC portal (https://portal.gdc.cancer.gov/). 10 RNA sequencing (RNA-seq) datasets were downloaded from Roadmap and TCGA, which are from the matched samples with methylomes. 69 ChIP-seq, 2 DNase-seq, 1 MNase-seq, and 1 Hi-C datasets were fetched from Roadmap or GEO. Source data for Fig 3 and Fig 4 are provided as Supplementary Data with the paper. Other data that support the findings of this study are available at https://github.com/JiejunShi/CAMDA/tree/master/paper-data.

Field-specific reporting

Please select the one below that is the best fit for your research. If you are not sure, read the appropriate sections before making your selection.

☒ Life sciences ☐ Behavioural & social sciences ☐ Ecological, evolutionary & environmental sciences

For a reference copy of the document with all sections, see nature.com/documents/nr-reporting-summary-flat.pdf

Life sciences study design

All studies must disclose on these points even when the disclosure is negative.

Sample size	Since only public available data are used for this study, we did not perform sample size calculations. The sample size is determined by the data availability.
Data exclusions	To reduce false positives, we only retained CpGs covered by at least four reads in the bisulfite sequencing data.
Replication	All results have been repeated or validated for at least two times in different datasets if multiple datasets are available. Results are consistent between repeated analysis.
Randomization	Samples are selected randomly if the whole dataset is not used.
Blinding	Since only public available data are used, this is not applicable.

Reporting for specific materials, systems and methods

We require information from authors about some types of materials, experimental systems and methods used in many studies. Here, indicate whether each material, system or method listed is relevant to your study. If you are not sure if a list item applies to your research, read the appropriate section before selecting a response.

Materials & experimental systems

n/a	Involved in the study	
☒	☐	Antibodies
☒	☐	Eukaryotic cell lines
☒	☐	Palaeontology and archaeology
☒	☐	Animals and other organisms
☒	☐	Human research participants
☒	☐	Clinical data
☒	☐	Dual use research of concern

Methods

n/a	Involved in the study	
☒	☐	ChIP-seq
☒	☐	Flow cytometry
☒	☐	MRI-based neuroimaging
